# Mechanisms and effects of metformin on skeletal muscle disorders

**DOI:** 10.3389/fneur.2023.1275266

**Published:** 2023-10-19

**Authors:** Ren Shang, Jing Miao

**Affiliations:** Department of Neurology, The First Hospital of Jilin University, Changchun, China

**Keywords:** metformin, inflammation, autophagy, mitochondrial biogenesis, skeletal muscle disorders

## Abstract

Skeletal muscle disorders are mostly genetic and include several rare diseases. With disease progression, muscle fibrosis and adiposis occur, resulting in limited mobility. The long course of these diseases combined with limited treatment options affect patients both psychologically and economically, hence the development of novel treatments for neuromuscular diseases is crucial to obtain a better quality of life. As a widely used hypoglycemic drug in clinical practice, metformin not only has anti-inflammatory, autophagy-regulating, and mitochondrial biogenesis-regulating effects, but it has also been reported to improve the symptoms of neuromuscular diseases, delay hypokinesia, and regulate skeletal muscle mass. However, metformin’s specific mechanism of action in neuromuscular diseases requires further elucidation. This review summarizes the evidence showing that metformin can regulate inflammation, autophagy, and mitochondrial biogenesis through different pathways, and further explores its mechanism of action in Duchenne muscular dystrophy, statin-associated muscle disorders, and age-related sarcopenia. This review clarifies the directions of future research on therapy for neuromuscular diseases.

## 1. Introduction

Skeletal muscle disorders are predominantly genetic in origin, among which are a number of rare diseases. The long disease courses and limited treatment methods greatly compromise patients’ quality of life, causing a heavy psychological burden. The economic pressure on families and society is also incalculable. Duchenne muscular dystrophy (DMD) is caused by a genetic defect leading to impaired dystrophin synthesis in muscle. As the disease progresses, patients’ mobility becomes severely limited, ultimately resulting in respiratory and circulatory failure (1). Furthermore, current treatment options for age-related muscle diseases and statin-associated muscle symptoms (SAMS) are unclear. Therefore, finding new treatment targets to alleviate the development process of these neuromuscular diseases and alleviate patient pain is crucial.

Metformin, a widely used hypoglycemic drug in clinical practice, originated from the traditional European herb, *Galega officinalis*, which was initially used to treat thirst and frequent urination. However, the side effects owing to lactic acidosis caused by other guanidines delayed its clinical usage (2, 3). Metformin was introduced into clinical practice following research evaluating its tolerance and long-term safety (3). Metformin inhibits gluconeogenesis through different pathways to lower blood glucose (4–6). Interestingly, along with its hypoglycemic effect, metformin can also improve skeletal muscle mass and strength through different mechanisms, thereby delaying disease progression. For example, it can exert its effects via regulating oxidative stress, calcium homeostasis, the expression of inflammatory factors, and improving mitochondrial dysfunction, to enhance the quality, strength, and regenerative ability of skeletal muscles.

Several studies have verified that metformin can improve the symptoms of neuromuscular diseases. The combined treatment of L-arginine and metformin has a beneficial impact on the decline of motor function and muscle degeneration of 7- to 10-year-old boys with DMD, and can delay disease progression (7). The combination of metformin and statins can also reduce statin-induced muscle spasms and pain symptoms (8). Furthermore, another study demonstrated that combinatorial treatment with metformin and leucine could improve muscle quality during aging (9). Moreover, our study found that metformin can restore damaged autophagic flux, enhance the vitality of fibroblasts from patients with UDP-N-acetylglucosamine 2-epimerase/N-acetylmannosamine kinase (GNE) myopathy, reduce the rate of apoptosis, and thus play a protective role in GNE myopathy (10). Due to the limited treatment methods available for skeletal muscle diseases, the above mentioned positive effects of metformin for their treatment is encouraging. Therefore, we summarized the mechanism of action of metformin in neuromuscular diseases to provide a useful resource for directing future research.

## 2. Pharmacokinetics of metformin

Metformin is a hydrophilic compound with a positive charge at physiological pH. The oral absorption, tissue distribution, and renal excretion of metformin are dependent on drug transporters. Metformin is a substrate for many organic cation transport proteins, such as OCTN1, OCTN2, PMAT, and multidrug and toxin extrusion transporters. OCT1 is the major transporter responsible for metformin uptake in the liver (11–13), wherein metformin is absorbed within 6 h after dosing, with a 50%–60% bioavailability. Approximately 70% of the dose is absorbed from the small intestine, while the rest enters the colon and is excreted in feces. Metformin is excreted via urine in its original form, with a half-life of approximately 5 h (14). The concentration of metformin accumulated in the jejunum is 30–300 times higher than that in plasma. The side effects reported at present mainly include gastrointestinal discomfort (including abdominal distension, abdominal pain, diarrhea or stomach pain, early satiety, loss of appetite, and nausea), but these side effects usually subside after 1–2 weeks of use (15). Other studies have found that some people taking metformin develop severe vitamin B12 deficiency, especially those over 80 years of age who are prone to this condition (16). This may be because metformin interferes with the absorption of vitamin B12 in the intestine and affects the balance of intestinal microorganisms (17). People with normal renal function generally have good tolerance to this drug; however, those with severe kidney, liver, or heart diseases might suffer from lactic acidosis (18).

## 3. Mechanism of action of metformin

### 3.1. Metformin and inflammation

**Figure 1:**
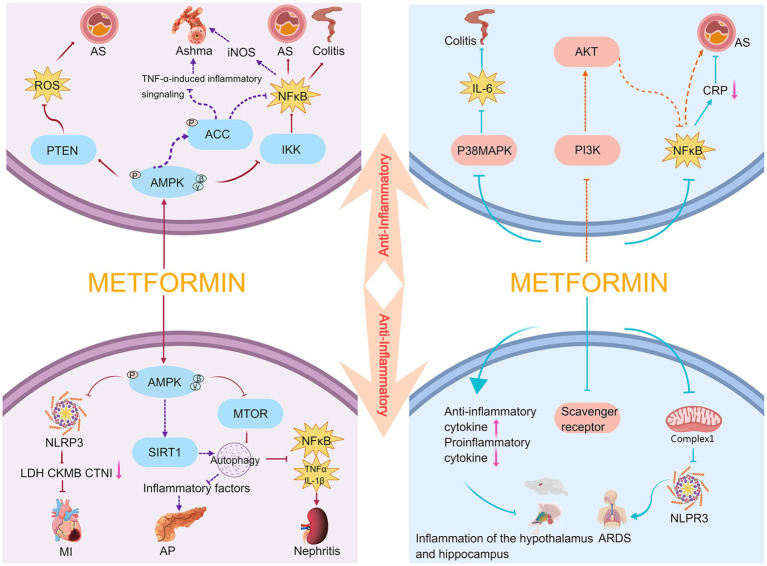
Anti-inflammatory mechanism of metformin. AS, atherosclerosis; AP, acute pancreatitis; MI, myocardial infarction.

Previous studies have illustrated the anti-inflammatory effects of metformin across animal models of disease and in human studies (Figure 1). Among them, nuclear factor kappa-light-chain-enhancer of activated B cells (NF-κB) plays an important role in the mediation of inflammation. NF-κB is a transcription factor composed of RelA (p65), c-Rel, RelB, NF-κB1 (p50), and NF-κB2 (p52); it has a significant impact on the regulation of cell adhesion, cytokine production, apoptosis, cell growth, and barrier function (19). NF-κB mediates the activation of inflammatory bodies, and its release can act as a trigger for the inflammatory cascade response (20). Its hyperactivation is associated with inflammation. Amp-activated protein kinase (AMPK) signaling can inhibit the inflammatory response induced by the NF-κB system (21). AMPK, as a serine/threonine protein kinase, plays a key role in energy metabolism and consists of three subunits: α subunits (α1, α2), regulatory β subunits (β1, β2), and γ subunits (γ1, γ2, γ3) (22). AMPK is targeted in a variety of inflammatory disease models, and metformin can inhibit the inflammatory response through AMPK-dependent and -independent pathways.

Macrophages are the body’s first line of defense against external pathogens and are crucial for triggering inflammation. To trigger the body’s defense response, macrophages secrete cytokines, chemokines, and growth factors; however, overproduction of these factors causes tissue damage and chronic inflammation. It has been reported that lipopolysaccharide induces an increase in chemokines in RAW264.7 cells (a mouse macrophage cell line), and metformin can inhibit NF-κB signaling by activating AMPK to exert its anti-inflammatory effects (23). Hattori et al. found that metformin activates AMPK and inhibits IκB kinase (IKK) activity to suppress NF-κB activation, thereby reducing the progression of vasculitis or atherosclerosis (24). Koh et al. reported that metformin may attenuate intestinal inflammation by inhibiting IKK/NF-κB signaling activation through AMPK (25). Park et al. and Calixto et al. also concluded that metformin significantly inhibits eosinophil inflammation and improves airway remodeling. These studies showed that metformin increases the levels of phosphorylated AMPK and its downstream target acetyl CoA carboxylase (ACC), and inhibits tumor necrosis factor-α (TNF-α)-induced inflammatory signaling and NF-κB-mediated inducible nitric oxide synthase expression in the lung tissue of obese mice (26, 27). Sun et al. found that metformin can regulate the autophagy and subsequent downregulation of inflammatory cytokines (including interleukin (IL)-1β and TNF-α), NF-κB expression, and ROS-induced apoptosis in diabetic/dyslipidemic (db/db) mice via the AMPK/mTOR signaling pathway to attenuate the renal inflammatory response and ameliorate abnormal renal pathology (28). Phosphatase and tensin homolog (PTEN) is a lipid and protein phosphatase whose attenuation promotes the inflammatory response. Kim et al. recently reported that metformin attenuates vasculitis and delays atherosclerosis development by negatively regulating ROS production through the AMPK/PTEN pathway (22).

The Nod-like receptor protein 3 (NLRP3) inflammasome comprises the sensor NLRP3, the adaptor protein ASC (apoptosis-associated speck-like protein containing a caspase recruitment domain), and the effector pro-caspase-1. The expression of critical components of the inflammasome and associated cytokines is upregulated by transcriptional activation, turning on the first step in the activation of the NLRP3 inflammasome (29). Recent studies have indicated that AMPK plays a key role in mediating the activation of the NLRP3 inflammasome (20, 30). Zhang et al. demonstrated that metformin exerts protective effects in a model of myocardial ischemia–reperfusion injury by reducing lactose dehydrogenase, creatine kinase MB, and cardiac troponin I through AMPK/NLRP3 and reducing myocardial inflammatory cell infiltration, myocardial collagen deposition, and the scope of myocardial infarction (31). Sirtuin 1 (SIRT1) exists in the nucleus and cytoplasm, and has nicotinamide adenine dinucleotide-dependent protein deacetylase activity (32). It has regulatory roles in various physiological functions, such as the cell cycle, cell metabolism, autophagy, mitochondrial function, oxidative stress, DNA repair, and inflammation. SIRT1 plays an important role when the body is under hypoxic or stressful conditions (33); further, it can be positively regulated by AMPK (34). In experimental acute pancreatitis models, both *in vivo* and *in vitro* experiments demonstrated that metformin restored impaired autophagy by upregulating SIRT1 expression mainly through AMPK activation, thereby reducing the release of inflammatory factors (35).

Overall, metformin can exert protective effects in atherosclerotic thrombotic disease (22, 24), intestinal inflammation (25), cardiovascular disease (31), asthma (26, 27), acute pancreatitis (35), and diabetic nephropathy (28) upon acting on the AMPK/NF-κB, AMPK/PTEN, AMPK/NLRP3, and AMPK/SIRT1 pathways. Metformin also inhibits inflammatory responses through AMPK-independent pathways. It inhibits the phosphorylation and nuclear translocation of the NF-κB subunit, p65, and inhibits the degradation of its inhibitory protein, IκB, resulting in NF-κB being isolated in the cytoplasm and unable to translocate to the nucleus to participate in the induction of inflammatory responses (36). In an atherosclerotic rabbit model, Li et al. demonstrated that metformin inhibits IκB phosphorylation and NF-κB activation, reduces serum C-reactive protein levels, and exerts anti-vascular inflammatory effects (37). Isoda et al. found that metformin ameliorates vasculitis by blocking NF-κB pro-inflammatory signaling through the PI3-K/Akt signaling pathway (38). Another study found that metformin inhibits the activation of the NLRP3 inflammasome and attenuates acute respiratory distress syndrome by targeting the electron transport chain complex 1 independent of the AMPK or NF-kB pathways (39).

In addition to the mechanisms described above, studies have reported that macrophages express several types of scavenger receptors which play an essential role in the inflammatory response. The interaction between scavenger receptors and oxidized low-density lipoprotein enhances NF-κB activity and produces pro-inflammatory cytokine production. Hyun et al. found that metformin downregulates the expression of scavenger receptors, CD36 and SR-A, to attenuate the inflammatory response in type 2 diabetic mice (40). Furthermore, Di Fusco et al. used metformin to treat mice with colitis and found that intestinal inflammation in mice was alleviated and accompanied by the activation of AMPK. However, neither activation nor the inhibition of AMPK expression affected the therapeutic effect of metformin on inflammation. Therefore, by exploring different pathways of metformin’s control on inflammation, they concluded that metformin could downregulate the activation of p38 MAP kinase in these mice through an AMPK-independent pathway and inhibit IL-6 expression (41).

Furthermore, cytokines are classified as either pro-inflammatory or anti-inflammatory. Pro-inflammatory cytokines, including IL-1, IL-6, IL-8, IL-12, TNF-α, and interferon, are secreted by Th1 cells, CD4+ T cells, macrophages, and dendritic cells. The key pro-inflammatory cytokines are IL-1, IL-6, and TNF-α. Anti-inflammatory cytokines include IL-4, IL-10, IL-11, IL-13, the IL-1 receptor antagonist, and TGF-β. The dynamic balance between pro- and anti-inflammatory cytokines plays a pivotal role in regulating inflammation (42). Additionally, Hammad et al. found that metformin decreased the relative mRNA expression of the pro-inflammatory factors IL-1β and IL-6, and increased the relative mRNA expression of the anti-inflammatory factor IL-10 in the hypothalamus and hippocampus, thereby decreasing inflammation in a rat model of oxandrolone-induced depressive-like behavior (43).

In summary, metformin can delay the progression of diseases such as vasculitis (37, 38), acute respiratory distress syndrome (39), type 2 diabetes-induced inflammatory response (40), colitis (41), and central nervous system inflammation (43) by blocking NF-κB inflammatory signaling, inhibiting NLRP3 inflammasome activation, suppressing the expression of scavenger receptors, and downregulating the expression of pro-inflammatory factors.

### 3.2. Metformin and autophagy

Autophagy, which was first proposed by the Belgian scientist, Christian de Duve, is one of the most investigated topics in cell biology. Also known as programmed cell death type II, the autophagic process is ubiquitous in nature, and is widely recognized as a cytoprotective mechanism (44). There are three main types of autophagy based on modes of cellular material transport to lysosomes (45, 46): (i) macroautophagy, (ii) microautophagy, and (iii) chaperone-mediated autophagy. We usually refer to macroautophagy as autophagy. The process of autophagy is divided into three stages: the formation of phagophores or isolation membranes, autophagosomes, and autolysosomes (47–49).

The formation of an autophagosome is a hallmark of autophagy initiation. Unc-51-like kinase 1 (ULK1), a molecule with serine/threonine kinase activity, is important in the formation of autophagosomes. When ULK1 is phosphorylated or ubiquitinated, it exerts an autophagy-inducing effect. During autophagy, Beclin-1 is involved in autophagosome membrane formation and material transport. ULK1 can phosphorylate Beclin-1 and VPS34; the activation of these targets is important for autophagy initiation. During autophagosome formation, microtubule-associated protein 1A/1B-light chain 3 (LC3) is processed into the soluble form, LC3-I. Then, LC3-I binds to phosphatidylethanolamine (PE) on the surface of the autophagosome membrane, becoming membrane bound LC3-II. Protein Sequestosome 1 (P62/SQSTM1) is expressed in a variety of cells and tissues, which can connect LC3 and its ubiquitination substrate as it integrates into an autophagosome and is degraded by autophagic lysosomes (50, 51). Autophagy is a dynamic process, and analyzing the expression of LC3 and P62 proteins can help us understand whether the flux of the autophagy is obstructed or not.

**Figure 2:**
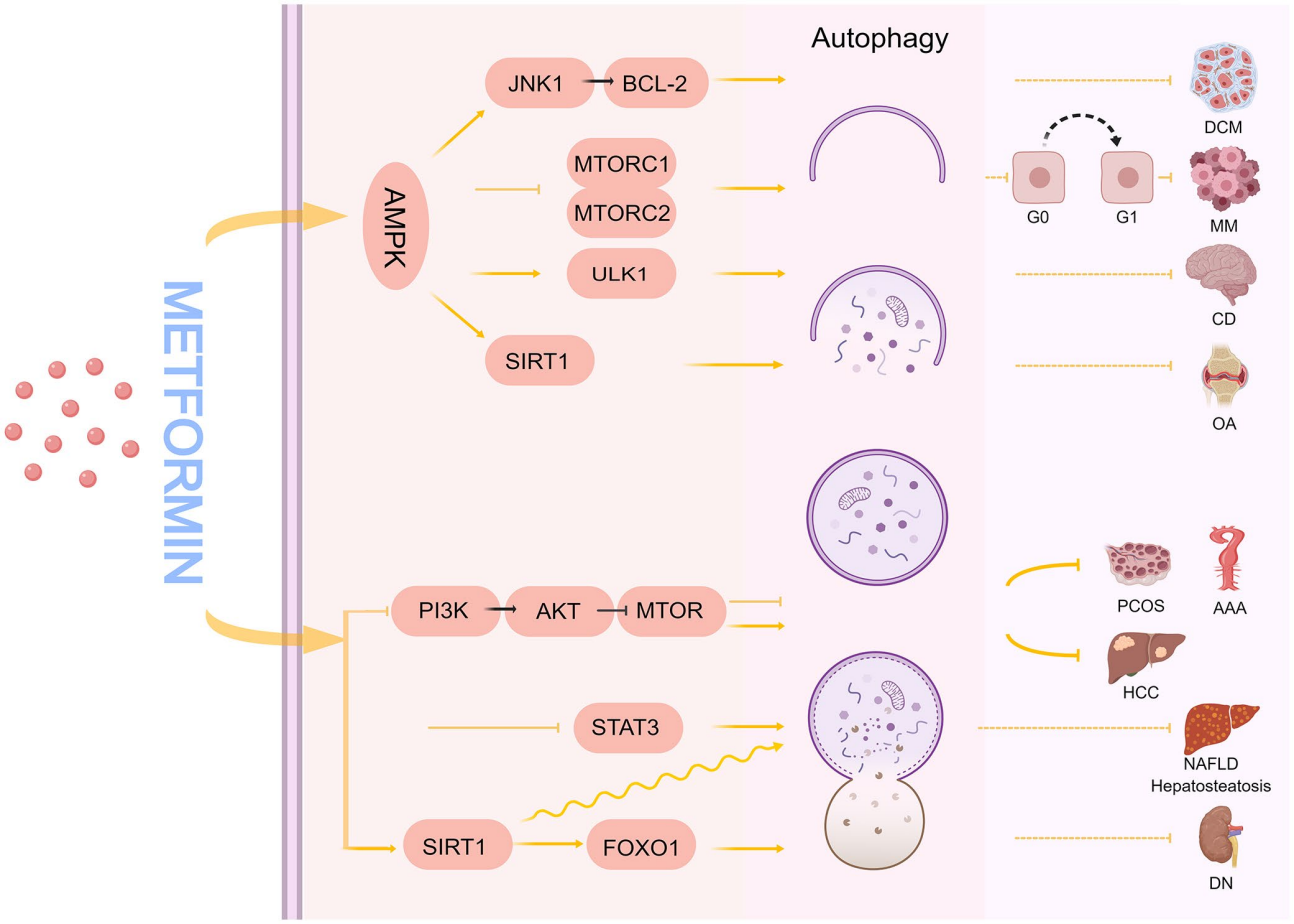
The mechanism of metformin regulating autophagy. DCM, diabetic cardiomyopathy; MM, multiple myeloma; CD, cognitive disorder; OA, osteoarthritis; HCC, hepatocellular carcinoma; PCOS, polycystic ovary syndrome; AAA, abdominal aortic aneurysm; NAFLD, non-alcoholic fatty liver disease; DN, diabetic nephropathy.

Under physiological conditions, autophagy remains low to maintain homeostasis. Hypoxia, nutrient deficiency, exposure to ROS, pathogen invasion, organelle damage, and excessive accumulation of abnormal proteins enhance autophagic activity (52). The process of autophagy involves several known and unknown signaling pathways. Metformin plays a vital role in regulating autophagy in many diseases (Figure 2), including polycystic ovary syndrome (PCOS) (53), tumors (54), cognitive disorders (55, 56), osteoarthritis (57), non-alcoholic fatty liver (58), hepatic steatosis (59), diabetic nephropathy (60), diabetic cardiomyopathy (61), and abdominal aortic aneurysm (62).

Many studies have demonstrated that metformin can regulate the autophagic process by relying on AMPK pathways, such as AMPK/mTOR, AMPK/ULK1, AMPK/SIRT1, and AMPK/JNK1/BCL-2. mTOR is an atypical serine/threonine protein kinase that plays a gateway role in autophagy (63); when mTOR is stimulated by nutrient, energy, and stress signals, it can promote or inhibit mTORC1/mTORC2 complex formation, which in turn regulates autophagy. In cancer, autophagy can act as a tumor suppressor (64, 65) or promoter (66) in different contexts and stages of cancer development. Wang et al. showed that metformin, as a potential specific dual mTORC1/2 inhibitor, downregulates p-mTOR (Se-2448), p-P70 S6K, p-4 EBP1, and p-AKT (Ser 473) expression through AMPK activation-mediated mTORC1/mTORC2 inhibition, induces autophagy, and causes cell cycle arrest in the G0/G1 phase of myeloma cells, which ultimately inhibits the growth of myeloma cells (54).

Autophagy regulates neuronal homeostasis in the central nervous system (67, 68). Defective autophagy may be a key driver of neurodegeneration, ultimately leading to neurocognitive impairment (69, 70). Li et al. found that 1.5% isoflurane caused impaired autophagy in the hippocampus of aged rats in a time-dependent manner, affecting spatial learning and memory (71). Mounting evidence indicates that autophagy upregulation has neuroprotective effects (55, 69). Zhu et al. found that 20-month-old mice (analogous to elderly humans) developed spatial learning and memory deficits with reduced autophagic activity when exposed to 3% sevoflurane for 2 h, and that autophagy deficits may have led to reduced microtubule-associated protein 2 (MAP2) and postsynaptic density protein 95 (PSD95) levels, as well as reduced synaptic density. Interestingly, another study found that metformin increased phosphorylation of AMPK (Thr172), which in turn caused increased phosphorylation of ULK1 (Ser317 and Ser777) and upregulated autophagic activity to protect against sevoflurane-induced synaptic decline and neurocognitive deficits, suggesting that metformin is useful in preventing or treating anesthesia-induced cognitive deficits in mice (55, 56).

SIRT1, a nicotinamide adenine dinucleotide-dependent deacetylase, is a key regulator of energy metabolism that significantly affects metabolism, inflammation, and autophagy regulation (72–74). It has been demonstrated that metformin can promote chondrocyte autophagy and reduce chondrocyte apoptosis through the AMPKα2/SIRT1 pathway to alleviate osteoarthritis, and the protective effect of metformin is reversed by silencing AMPKα2 or blocking SIRT1 expression (57). Furthermore, Beclin-1 binds to Vps34 to form a PI3K-Beclin-1 complex involved in the formation of the autophagic pre-membrane, and a growing body of data supports the hypothesis that Bcl-2 inhibits autophagy membrane generation by binding Beclin-1. Consequently, interference with Bcl-2 may result in the dissociation of Bcl 2/Beclin1, which will lead to increased cellular autophagy (75). He et al. found that metformin can activate AMPK to stimulate the JNK1-BCL2 signaling pathway, cause Bcl-2 and Beclin-1 dissociation, restore autophagy, and alleviate diabetic cardiomyopathy (61).

In addition to the AMPK pathway mentioned above, metformin can also regulate autophagy through AMPK-independent pathways. Among them, the PI3K/AKT/mTOR signaling pathway is associated with various functions such as cell proliferation, differentiation, metabolism, apoptosis, and autophagy (76, 77). Sun et al. found that aloin and metformin alone or in combination can induce apoptosis and autophagy, inhibit the growth and invasion of hepatocellular carcinoma, and enhance anti-tumor effects through the PI3K/AKT/mTOR pathway (78). The PI3K/AKT/mTOR signaling pathway can regulate autophagy in both directions (79, 80). One study demonstrated that in an angiotensin II (Ang-II)-induced ApoE−/− mouse abdominal aortic aneurysm model, metformin inhibited PI3K/AKT/mTOR pathway activation, and reduced Ang-II-induced autophagy-related protein levels to inhibit aortic dissection, thus, preserving aortic elastin structure and reducing collagen loss and aortic cell apoptosis (62). In PCOS, oxidative stress levels are significantly elevated, leading to the excessive activation of autophagy, while metformin reduces the excessive autophagy in ovarian granulosa cells through PI3K/AKT/mTOR targeting to ameliorate PCOS symptoms (53). In addition, metformin inhibits STAT3 mRNA and protein expression, activates autophagy, and reduces the production of inflammatory cytokines in non-alcoholic steatohepatitis disease models (58). Evidence also supports that metformin can regulate autophagy through an AMPK-independent pathway. For example, Song et al. found that SIRT1 and autophagy were significantly downregulated in the livers of ob/ob (obese) mice, and in a hepatocyte model induced by oleic acid and high glucose, metformin increased SIRT1 expression and induced autophagy through an AMPK-independent pathway to reduce hepatosteatosis in ob/ob mice (59). Xu et al. constructed a diabetic rat model through a high-fat diet combined with an intraperitoneal injection of streptozotocin. After 8 weeks of intraperitoneal metformin injections, they found that the glomerular structure and renal function of diabetic rats were significantly improved, and the primary mechanism was that metformin regulated the SIRT1/FOXO1 autophagic signaling axis to induce renal autophagy (60).

Vacuolar protein sorting 15 (VPS15), which is one of the components of the phosphatidylinositol 3-kinase (P13K) complex, contributes to the generation of autophagosomes and endosomes. In patients with Danon disease and glycogen storage disease type II, it was found that VPS15 expression is elevated (81), indicating autophagy disorders (81, 82). Nascimbeni et al. considered that the effects of early use of short to long term Enzyme Replacement Therapy can help restore autophagic flux, enhance skeletal muscle mass, and enhance acid α-glucosidase enzyme activity (83). Therefore, it is speculated that metformin may be helpful in restoring autophagic flux in this disease. However, further research is needed to confirm these findings.

### 3.3. Metformin promotes mitochondrial biogenesis

Mitochondrial biogenesis is the intracellular synthesis of new healthy mitochondria, which can be activated by different signals under stressful conditions (84, 85). Mitochondrial biogenesis is related to cell division, oxidative stimulation, increased cellular energy demand, and exercise training. Some diseases and other factors can also affect mitochondrial biogenesis, including the pathogenesis of Huntington’s, Alzheimer’s, and Parkinson’s disease (86, 87).

**Figure 3:**
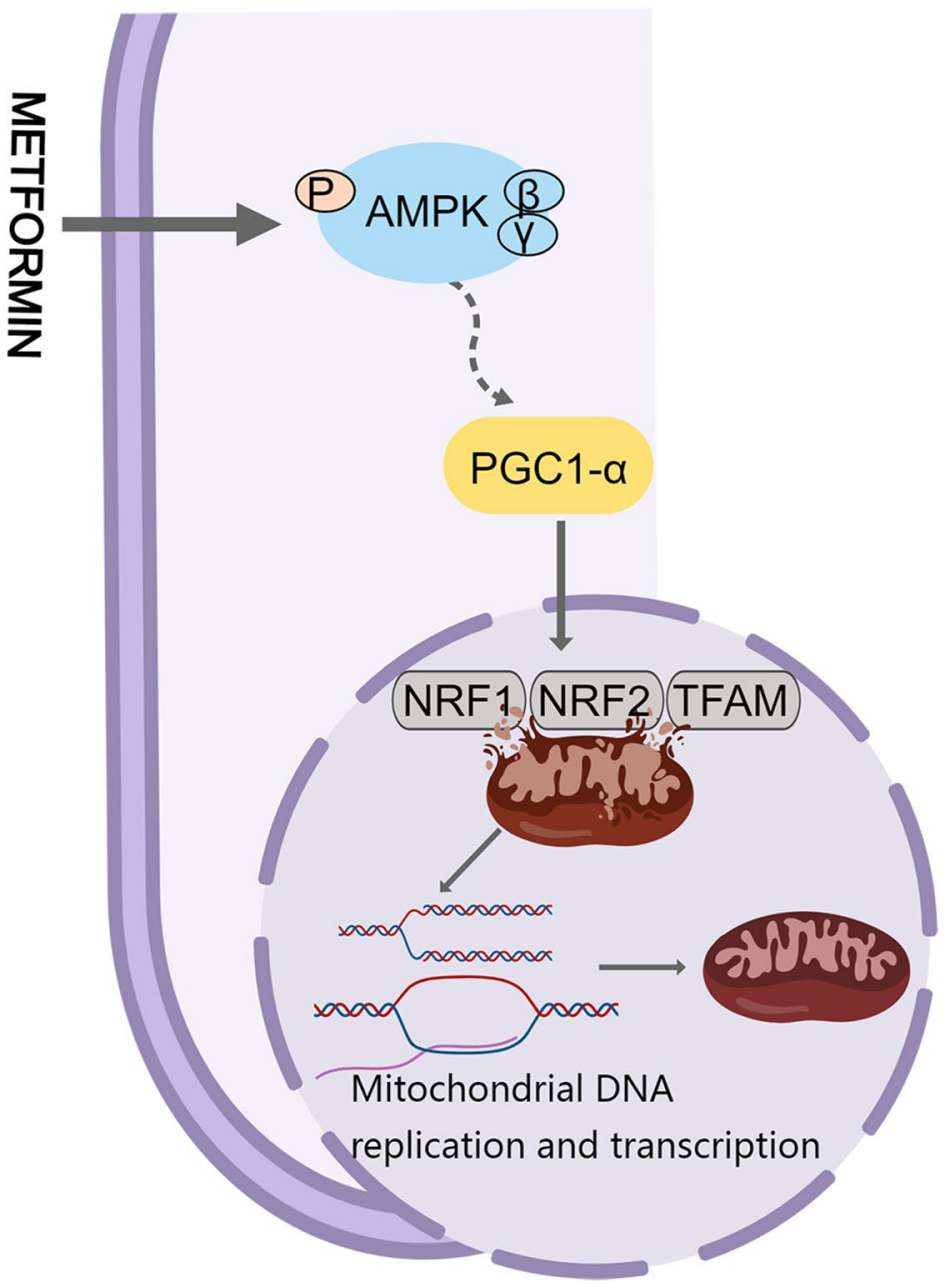
Metformin and mitochondrial biogenesis. PGC-1α, Peroxisome proliferator-activated receptor-γ co-activator-1α.

AMPK is considered a key regulator in initiating mitochondrial biogenesis (88). Cells can regulate mitochondrial biogenesis through Peroxisome proliferator-activated receptor-γ co-activator-1α (PGC-1α), estrogen receptors, and transcription factors. PGC-1α is a major regulator of mitochondrial biogenesis. AMPK stimulates PGC-1α phosphorylation, which triggers the expression of transcription factors involved in mitochondrial biogenesis (nuclear respiratory factor (NRF)-1 and NRF-2) and mitochondrial transcription factor A (TFAM) (89, 90), thereby promoting mitochondrial DNA replication and transcription (Figure 3). The levels of PGC-1α, NRF-1, NRF-2, and TFAM proteins were reduced in a mouse model of peritoneal fibrosis associated with peritoneal dialysis, indicating significant inhibition of mitochondrial biosynthesis. Metformin may partially act on the AMPK/PGC-1α pathway to increase mitochondrial DNA content, improve mitochondrial morphology, and enhance mitochondrial biosynthesis to reduce peritoneal dialysis related peritoneal fibrosis in mice (91).

In the central nervous system, neuronal survival is compromised when mitochondria are damaged (92). The function or expression of PGC-1α is impaired to different degrees in degenerative diseases. Remarkably, overexpression of PGC-1α reduces mitochondrial damage and improves biogenesis (93). Khang et al. found that metformin exerts neuroprotective effects against Parkinson’s disease by reversing the reduction of Parkin in the substantia nigra and the accumulation of parkin-interacting substrate, and by downregulating the expression of PGC-1α in db/db mice and mice fed a high-fat diet (94). Metformin markedly improves aging-induced cardiomyocyte dysfunction (95). Gundewar et al. found that administration of 125 mg/kg metformin to a mouse model of heart failure resulted in AMPK activation, increased PGC-1α expression, and increased phosphorylation of endothelial nitric oxide synthase, which in turn improved mitochondrial function and significantly reduced ventricular dysfunction and improved survival (96). To sum up, metformin may have therapeutic potential in treating peritoneal fibrosis, neurodegenerative diseases, and cardiovascular diseases by modulating the quality of the mitochondria.

## 4. Metformin and skeletal muscle disorders

### 4.1. DMD

#### 4.1.1. Clinical manifestations of DMD

Mutations in the Dystrophin gene result in dystrophinopathies, such as DMD, Becker muscular dystrophy, and others. Approximately 75% of Dystrophin gene mutations are intragene deletions (65%) or duplications (10%), with the remaining 25% being nucleotide variants (97). One of the most severe phenotypes is DMD, an X-linked recessive neuromuscular disorder. About 1 in 5,000 people suffer from DMD (97). Muscle fiber cells rely on dystrophin to maintain their functionality by linking the intracellular cytoskeleton with the transmembrane components of the dystrophin-glycoprotein complex (98). In the absence of dystrophin, the cytoskeleton and sarcolemma fail to connect properly, resulting in damage during muscle contraction, leading to chronic inflammation which impairs muscle fiber myogenesis and regeneration (99, 100). In DMD, delayed milestones in motor development during early childhood at the age of 4–5 years, typical Gowers syndrome, limited mobility, and pseudohypertrophy of calf muscles can occur (101). As the disease progresses, abnormal posture and gait may develop (102). Myocardial involvement develops in severe cases. Over time, it may even progress into dilated cardiomyopathy (1). Ultimately, death occurs by 20–30 years of age owing to respiratory and circulatory failure (103). Glucocorticoid therapy would significantly delay the decline of muscle strength and function, however, side effects such as obesity, cushingoid facies, and osteoporosis should be considered. In addition, deflazacort, Debio-025, and vamorolone can also improve the symptoms of DMD to varying degrees. Angiotensin-converting enzyme inhibitors, angiotensin receptor blockers, and β-blockers are the first choice for patients with cardiac involvement. The gene therapy strategies for DMD include gene transfer, exon reframing, exon skipping, exon reconstruction, and CRISPR gene editing. The latter three as personalized treatment options are beneficial for only a small number of patients (97).

#### 4.1.2. Mechanism of metformin in the treatment of DMD

A clinical study showed that combined treatment with L-arginine and metformin positively affected hypokinesia and muscle degeneration in boys aged 7–10 years with DMD (7). Ljubicic et al. demonstrated that 42 days of metformin treatment of mdx mice resulted in increased expression of utrophin A, a compensatory functional target for the lack of dystrophin (104). The neuromuscular junction (NMJ), which connects motor neurons to skeletal muscle fibers, plays a crucial role in controlling muscle contractions (1). Fragmentation of NMJs has been observed in DMD patients and mdx mouse models (105). A previous study suggested that the NMJ in mdx mice show decreased levels of muscle specific kinase (MuSK) (106); this deficiency results in abnormal NMJ morphology in mdx mice as it plays an important role in acetylcholine receptor aggregation. The reduction of MuSK in mdx mice disrupts the normal latticed network of microtubules, potentially causing disruption of the resting NMJ structure. Dong et al. discovered that metformin increased muscle strength and improved muscle membrane integrity and NMJ transmission in *mdx* mice (1). All these studies demonstrate that metformin is a highly potent drug for treating DMD.

AMPK is essential for the normal tilting process of macrophages, which is required for normal muscle regeneration; it further plays an invaluable role in maintaining and remodeling skeletal muscle (107). For example, in myopathies caused by mitochondrial diseases, AMPK activator (AICAR)-induced AMPK activation notably attenuated the pathology, mainly by promoting muscle regeneration (108). Apart from this, Thomase et al. found that double knockout of AMPKβ1 and AMPKβ2 in mice produced a myopathic phenotype with a marked increase in the number of myofibers in the central nucleus, an abnormal distribution of myofibers, and many opaque fibers (109). Wang et al. argue that deletion of the AMPKα1/2 subunit decreases mitochondrial density and membrane potential. Further, using metformin in primary hepatocytes prepared from AMPKα1/2-knockout mice did not improve the mitochondrial membrane potential or density, suggesting that metformin increases mitochondrial density through an AMPK-dependent pathway (110). Metformin activates AMPK primarily by inhibiting mitochondrial electron transport chain complex I and regulating AMP/ATP and/or ADP/ATP ratios, which regulates mitochondrial biogenesis and function (111). The activation of AMPK plays a crucial role in maintaining skeletal muscle function. AMPK activity and phosphorylated AMPK levels in skeletal muscle were increased by approximately 80% from baseline levels in humans taking metformin (2 g/day) for approximately 10 weeks (112).

Ca^2+^, the main regulator and signaling molecule in skeletal muscle, acts as a messenger in processes ranging from contraction activation to myocyte degradation. A sophisticated system of controlling Ca^2+^ concentration in the cytoplasm exists in skeletal muscle (113). Muscle calcium overload usually occurs in myodystrophy, at which point calpain is activated, leading to the proteolytic hydrolysis of cellular components (114). Cardiotoxins initiate muscle damage, inflammation, and myofilament destruction, similar to the pathology of myodystrophy (115). Through *in vitro* and *in vivo* experiments, Langone et al. demonstrated that metformin most likely reduces muscle necrosis by negatively regulating the inward flow of Ca^2+^ (114).

In DMD, due to the absence of muscular dystrophy protein, matrix metalloproteinase-9 (MMP-9) expression is upregulated, leading to the disruption of the connection between the extracellular matrix and cell membrane in skeletal muscle (116). MMP-9 is a biomarker associated with pathological progression of muscle injury and malnutrition, and increased expression of MMP-9 may be involved in muscle inflammatory responses (117). A recent study found that inhibiting MMP-9 gene expression can improve muscle function and reduce inflammation and fiber necrosis in mdx mice (118). TGF β-1 as a key fibrogenic biomarker positively correlated with the skeletal muscle pathology of mdx mice. MMP-9 is an activator of latent TGFβ-1 in skeletal muscle. Mantuano et al. found that metformin can significantly reduce TGFβ-1 levels to reduce pathological changes in mdx mice (111).

In general, the evidence supports the notion of a beneficial role of metformin in maintaining skeletal muscle function and treating DMD through the modulation of AMPK or skeletal muscle Ca^2+^ distribution or TGFβ-1 levels. Therefore, metformin may be a highly promising agent for the treatment of DMD.

### 4.2. Statin-associated myopathy

#### 4.2.1. Clinical manifestations of statin-associated myopathy

Statins are inhibitors of hydroxymethylglutaryl-coenzyme A (HMG-CoA) reductase, the key enzyme inhibiting cholesterol biosynthesis, and are widely used in treating patients with dyslipidemia. Muscle toxicity is a rare adverse effect associated with statin therapy, and clinical symptoms are related to skeletal muscle discomfort, ranging in severity from mild muscle stiffness or pain to severe life-threatening rhabdomyolysis (119, 120). After discontinuation of medication, it can also be accompanied by persistent myalgia and elevated serum creatine kinase (CK) (121). At present, the best method to manage statin related muscle diseases is to prevent them in advance, use the lowest dose of statin drugs as possible to achieve therapeutic effects, avoid combining them with other drugs that can increase the risk of muscle disease, and guide patients to pay attention to whether muscle discomfort symptoms occur when taking medication (122). Skeletal muscle biopsy reveals myonecrosis or even rhabdomyolysis. Unfortunately, the muscle pain caused by statins has led many patients to stop or refuse to take the drug.

#### 4.2.2. Mechanism of metformin in the treatment of statin-associated myopathy

Multiple lines of evidence indicated that SAMS pathogenesis may be due to statin-associated mitochondrial dysfunction (123–126). Increased Atrogin-1 in muscle tissue (127) causes increased skeletal muscle proteolysis. Mallinson et al. observed an increase in Atrogin-1 mRNA expression in muscles associated with mitochondrial dysfunction during patient treatment with statins (128). Type II muscle fibers with low mitochondrial content have long been considered more susceptible to the effects of statin therapy (119). An 8- to 40-fold increase in Atrogin-1 mRNA was found in different types of muscle atrophy (129), and the increase in Atrogin-1 expression preceded the decrease in muscle weight (130). FOXO3 is a crucial factor for the induction of Atrogin-1 (129). Sandri et al. showed that overexpression of PGC-1α inhibited the rapid loss of muscle mass induced by FOXO3. PCG-1α appears to exert this protective effect by repressing the transcription of *FOXO3* via genes involved in muscle atrophy (131). One study found that overexpression of PGC-1α significantly ameliorated lovastatin-induced muscle damage and reduced Atrogin-1 expression in zebrafish and mouse myotubes. Therefore, it is speculated that metformin, a drug that increases the action or activity of PGC-1α, may prevent or counteract the deleterious effects of statins on muscle (127).

There are relatively few studies on metformin and SAMS, and the mechanisms by which metformin improves the progression of SAMS and the role of mitochondria in the disease still need to be further explored.

### 4.3. Age-related sarcopenia

#### 4.3.1. Clinical manifestations of age-related sarcopenia

Aging is an inevitable part of life, and with advancing age, skeletal muscles experience a decrease in muscle mass, strength, and regenerative capacity (132–134). The decline in skeletal muscle mass and function caused by aging is also defined as sarcopenia.

The gradual loss of muscle mass causes a decline in both strength and function of the tissues, and reduced mobility leads to a decline in quality of life. Sarcopenia and its complications are a major threat to the health of the elderly. Starting from the age of 50, muscle mass decreases by 1–2% annually (135), the probability of developing sarcopenia is approximately 6–22% for individuals aged 60 to 70 years, and as high as 50% for individuals over 80 years of age (136). With the development of society and the pursuit of quality of life of older adults, it is urgent to overcome the damage to muscle structure and function caused by aging.

#### 4.3.2. Mechanism of metformin in age-related sarcopenia

Among the underlying factors of age-related muscle atrophy are reduced mitochondrial function, impaired function of satellite cells, and increased expression of inflammatory factors (137–139). Mitochondria play a crucial role in maintaining normal cellular function and skeletal muscle bioenergetics. Numerous findings support the notion that mitochondrial content is age dependent. For instance: (a) aging leads to a decrease in the mitochondrial number, density, and size; (b) mitochondrial DNA and protein expression decline (140–143); and (c) mitochondrial function decline occurs (143–145). PGC-1α, a target for protecting skeletal muscle atrophy, downregulates FOXO3 to prevent muscle atrophy (131). Cannavinoa et al. found that the overexpression of PGC-1α blocks MuRF-1 and Atrogin-1 induction, and activates autophagy to exert protective muscle function, and compounds that stimulate PGC-1α expression that can be used to treat or prevent muscle atrophy (146). This may provide novel guidance for the clinical treatment of muscular dystrophy myopathy. Hasan et al. reported that the modulation of the PGC-1α/FOXO3 signaling pathway by metformin improved high-fat diet-induced myofiber atrophy and fibrosis (147). Therefore, modulating PGC-1α might be beneficial in preventing age-related muscle atrophy.

It is reported that as age increases, the loss of innervation of motor neurons and muscle fibers leads to an increase in fast switch myofibers loss, leading to a decrease in muscle mass and strength (148, 149). Tezze et al. found that the combination of metformin and galantamine (RJx-01) can prolong the lifespan, exercise ability, and beneficial effects on muscle structure and function of *C. elegans*. Moreover, Rjx-01 treatment effectively increased the fast glycolytic fibers in Opa1−/− mice (characterized by a generalized aging phenotype) (150). Mitochondrial dysfunction and mitochondrial quality control system disorder are two of the factors leading to sarcopenia, fragmentation or abnormal enlargement of mitochondria in aging skeletal muscles of rodents or humans, Mitochondrial dysfunction leads to increased expression of mitogenes from muscles (FGF21 and GDF15) and impaired mitochondrial activity (151). In aged mice treated with RJx-01, there was a significant improvement in the number of mitochondrial cristae, electron particle matrix, a swollen appearance, and levels of FGF21 and GDF15 compared to the untreated group (150).

Muscle stem cells, also known as satellite cells, usually remain stationary. Satellite cells are essential in the process of aging muscle disuse and recovery. As age increases, the number and function of satellite cells decrease, and the stationary state of satellite cells in aging skeletal muscles is disrupted, resulting in damage to the cell quality control system and inability to maintain organelles and protein homeostasis. A previous study showed that aging satellite cells exhibit autophagy disorders, leading to a decrease in the number and function of satellite cells. Using rapamycin to induce autophagy can prevent satellite cells from aging and restore their regenerative ability (152). Tezze et al. also found that the levels of the transcription factor Pax7 (a marker of satellite cells) increased in aged mice treated with RJx-01, which is beneficial for satellite cell renewal (150). Another study found that the dual treatment of metformin and leucine can improve muscle quality during aging, mainly by increasing the content of satellite cells and reducing the accumulation of collagen, thus increasing the contractible tissue per cross-sectional area of muscle (9).

Inflammation is also one of the causes of sarcopenia. During the aging process, pro-inflammatory cytokines increase, inducing a chronic low-grade inflammatory state in the body. Due to the increase in pro-inflammatory cytokines, muscle strength in the elderly is significantly impaired (153, 154). Tezze et al. found that RJx-01 treatment of Opa1−/− mice can reduce the expression of inflammatory factors (IL-6, IL-1α and IL-1β) in plasma and skeletal muscle (150). Another study found that metformin may play a protective role in preventing sarcopenia by activating AMPK and reducing the expression of pro-inflammatory factors (155). It has been suggested that aging leads to impaired macrophage function (156). There are two types of macrophages: pro-inflammatory type M1 and anti-inflammatory type M2. M2 macrophages prevent muscle atrophy and promote muscle recovery (157). Long et al. found that 10-weeks of metformin treatment can increase the abundance of M2 macrophages and reduce the expression of inflammatory factor genes in muscle tissue (158).

In the future, the molecular and cellular underpinnings of mitochondrial regulation, muscle satellite cells, and inflammatory regulation should be investigated to explore the mechanisms by which metformin improves age-related skeletal muscle atrophy and functional decline to prolong and improve the quality of life of older patients.

## 5. The negative effects of metformin on skeletal muscle disorders

There are differing opinions on the role of metformin in skeletal muscle diseases (Table 1). One study found that, after administering 1,700 milligrams of metformin (*N* = 46) or placebo (*N* = 48) to patients who underwent 14 weeks of progressive resistance exercise training, metformin had a negative impact on the increase in skeletal muscle mass and strength caused by progressive resistance exercise training (159). Kang et al. believe that metformin decreases skeletal muscle mass by regulating the expression of myostatin through the AMPK-FOxO3a-HDAC6 axis (160). In another study, it was found that long-term use of low-dose metformin (0.1% w/w in diet) can prolong the lifespan of mice, but higher doses (1% w/w) may significantly shorten the lifespan due to lactic acid poisoning and renal failure (161). Campagnoli et al. found that oral metformin (1,500 mg/day) can cause a decrease of sex hormone levels in breast cancer patients without diabetes (162); however, drastic changes in hormones can easily lead to the degradation of skeletal muscle mass, increase the risk of sarcopenia, and to some extent, cause a decrease in lean mass (163). Therefore, the role of metformin in the regulation of skeletal muscle mass still needs further exploration, and hormone levels and dose dependence are issues that need further consideration.

**Table 1:** Mechanisms and effects of metformin on skeletal muscle disorders.

		Dose	Mechanism of action of metformin	Refs
DMD	Positive effect	Combination therapy of 2,500 mg of l-citrulline and 250 mg of metformin, 3 times daily	Significantly reduced muscle degeneration and improved motor function	(7)
		2 mg/mL	Increased expression of utrophin A	(104)
		200 mg/kg/day	Increased muscle strength, improved muscle membrane integrity and neuromuscular junction transmission	(1)
		200 mg/kg/day	Reduced muscle necrosis by negatively regulating the inward flow of Ca2+	(114)
		200 mg/kg/day	Reduced TGF β-1 expression	(111)
Statin-associated myopathy	Positive effect	-	Reduced Atrogin-1 expression	(127)
Age-related sarcopenia	Positive effect	320 mg/day	Improved muscle fiber atrophy and fibrosis partially through PGC-1 α/FOXO3	(147)
		410 mg/kg/day Met and 3.28 mg/kg/day Gal	Increased the fast glycolytic fibers	(150)
			Improved mitochondrial morphology and mitochondrial activity	
			Increased transcription factor Pax7	
			Reduced the expression of inflammatory factors	
		336.6 mg/kg/day ± 7.5 SE	Increased the content of satellite cells and reduced the accumulation of collagen, thus increasing the contractible tissue per cross-sectional area of muscle	(9)
		1,700 mg/day	Reduced the expression of inflammatory factor genes in muscle tissue	(158)
Muscle hypertrophy	Negative effect	1,700 mg/day	Decreased skeletal muscle mass and strength	(159)
Muscle atrophy		250 mg/kg, three times a week	Decreased skeletal muscle mass by regulating myostatin expression through the AMPK-FOxO3a-HDAC6 axis	(160)

## 6. Conclusion and perspectives

To summarize, although there are some controversies regarding the role of metformin in skeletal muscle diseases, numerous cell culture studies, animal studies, and clinical trials have shown that the beneficial effects of metformin on inflammation, autophagy, and mitochondrial function give us great confidence in its ability to improve skeletal muscle atrophy and delay disease progression. These influential reports further advance our understanding of the molecular mechanisms by which metformin exerts multiple biological activities. In the future, focusing on the molecular signaling mechanisms involved in the multiple activities of metformin should provide additional directions for the treatment of neuromuscular diseases.

## Author contributions

RS: Conceptualization, Investigation, Visualization, Writing – original draft. JM: Conceptualization, Project administration, Supervision, Writing – review & editing.
